# Determination of Leuprolide–Fatty Acid Conjugate in Rat Plasma Using LC-MS/MS and Its Pharmacokinetics after Subcutaneous Administration in Rats

**DOI:** 10.3390/molecules27248716

**Published:** 2022-12-09

**Authors:** Gi-Sang Seong, Seong-Wook Seo, Ji Young Cho, Kye Wan Lee, Beom-Jin Lee, In-Soo Yoon, Hyo-Eon Jin

**Affiliations:** 1 College of Pharmacy, Ajou University, Suwon 16499, Republic of Korea; 2 Department of Manufacturing Pharmacy, College of Pharmacy and Research Institute for Drug Development, Pusan National University, Busan 46241, Republic of Korea; 3 Dongkook Pharmaceutical Co., Ltd., Seoul 06072, Republic of Korea; 4 Research Institute of Pharmaceutical Science and Technology, Ajou University, Suwon 16499, Republic of Korea

**Keywords:** comparative pharmacokinetics, fatty-acid-conjugated leuprolide, leuprolide, peptide, UPLC-MS/MS

## Abstract

Leuprolide is a synthetic nonapeptide drug (pyroGlu-His-Trp-Ser-Tyr-d-Leu-Leu-Arg-Pro-NHEt) that acts as a gonadotropin-releasing hormone agonist. The continuous administration of therapeutic doses of leuprolide inhibits gonadotropin secretion, which is used in androgen-deprivation therapy for the treatment of advanced prostate cancer, central precocious puberty, endometriosis, uterine fibroids, and other sex-hormone-related conditions. To improve the pharmacokinetic properties of peptide drugs, a fatty acid was conjugated with leuprolide for long-term action. In this study, we developed a simple ultra-performance liquid chromatography-tandem mass spectrometry (UPLC-MS/MS) method for the simultaneous determination of leuprolide and leuprolide–oleic acid conjugate (LOC) levels. The developed method was validated in terms of linearity, precision, accuracy, recovery, matrix effect, and stability according to the US Food and Drug Administration guidelines, and the parameters were within acceptable limits. Subsequently, the pharmacokinetics of leuprolide and LOCs were evaluated. In vivo rat subcutaneous studies revealed that conjugation with fatty acids significantly altered the pharmacokinetics of leuprolide. After the subcutaneous administration of fatty-acid-conjugated leuprolide, the mean absorption time and half-life were prolonged. To the best of our knowledge, this is the first study showing the effects of fatty acid conjugates on the pharmacokinetics of leuprolide using a newly developed UPLC-MS/MS method for the simultaneous quantification of leuprolide and LOCs.

## 1. Introduction

Gonadotropin-releasing hormone (GnRH) is a decapeptide (pyroGlu-His-Trp-Ser- Tyr-Gly-Leu-Arg-Pro-Gly-NH_2_) that plays important roles in both the neural and endocrine systems. It is synthesized and stored in the medial basal hypothalamus [1]. It acts on GnRH receptors to signal both the synthesis and secretion of gonadotropin hormones, including luteinizing and follicle-stimulating hormones [1]. Leuprolide is a synthetic nonapeptide drug (pyroGlu-His-Trp-Ser-Tyr-d-Leu-Leu-Arg-Pro-NHEt) that acts as a GnRH agonist [2]. Continuous administration of therapeutic doses of leuprolide inhibits gonadotropin secretion, which is used in androgen-deprivation therapy for the treatment of advanced prostate cancer, central precocious puberty, endometriosis, uterine fibroids, and other sex-hormone-related conditions [3,4,5,6,7,8,9,10,11].

Peptide drugs, such as leuprolide, have a short plasma half-life because they have poor physicochemical stability in plasma and are rapidly eliminated by the kidneys [12]. To improve the pharmacokinetic properties of peptide drugs, various approaches have been applied to leuprolide for long-term action. The use of biodegradable polymeric nano/microparticles was proposed for the formulation of leuprolide [13]. Leuprolide is adsorbed onto poly(d,l-lactide-co-glycolide) (PLGA) nanoparticles and infiltrated into porous PLGA microspheres by dipping the structures into microsphere suspension. As a result, leuprolide is adsorbed onto both the surfaces of the nanoparticles and the microspheres, showing a slower release rate. Hu et al. [14] covalently linked hydrolyzable ester linkages on leuprolide to form polymeric micelles. The results showed that this approach significantly increased the circulation half-life of leuprolide when the drug covalently linked to the polymeric micelles was administered via intravenous injection. Kim et al. [15] developed an injectable liquid-crystal-forming system (LCFS) using sorbitan monooleate mixed with phosphatidylcholine, tocopherol acetate, and leuprolide acetate for a sustained-release injection. Compared to a commercial depot formulation of leuprolide, LCFS showed a similar area-under-the-curve (AUC) value and significantly reduced initial burst with a sufficient suppression of testosterone after subcutaneous injection in rats and dogs.

Drug modification via conjugation has also been applied to leuprolide. Recently, Fu et al. [16] improved the pharmacokinetic properties of leuprolide via polyethylene glycol (PEG)ylation. Two types of PEG, 2000 and 5000 PEG, were used for the PEGylation of leuprolide. PEGylation significantly increased the serum levels of testosterone compared to those in the control and leuprolide-only groups. Recently, we designed leuprolide-fatty acid conjugates to increase the pharmacokinetic stability of leuprolide [17]. In this study, we aimed to develop a bioanalytical LC-MS/MS method for leuprolide and its conjugate. Leuprolide was conjugated with oleic acid (C18), known as leuprolide–oleic acid conjugates (LOCs). This study aimed to establish a highly sensitive bioanalytical method to measure leuprolide and LOC concentrations in the plasma collected from Sprague–Dawley (SD) rats. We further investigated the quantitative performance of the liquid chromatography-tandem mass spectrometry (LC-MS/MS) system used to simultaneously quantify leuprolide and LOCs. We applied the established and validated method for the pharmacokinetic study of subcutaneous leuprolide and LOC administration in rats.

## 2. Results and Discussion

### 2.1. Method Development

**Figure 1:**
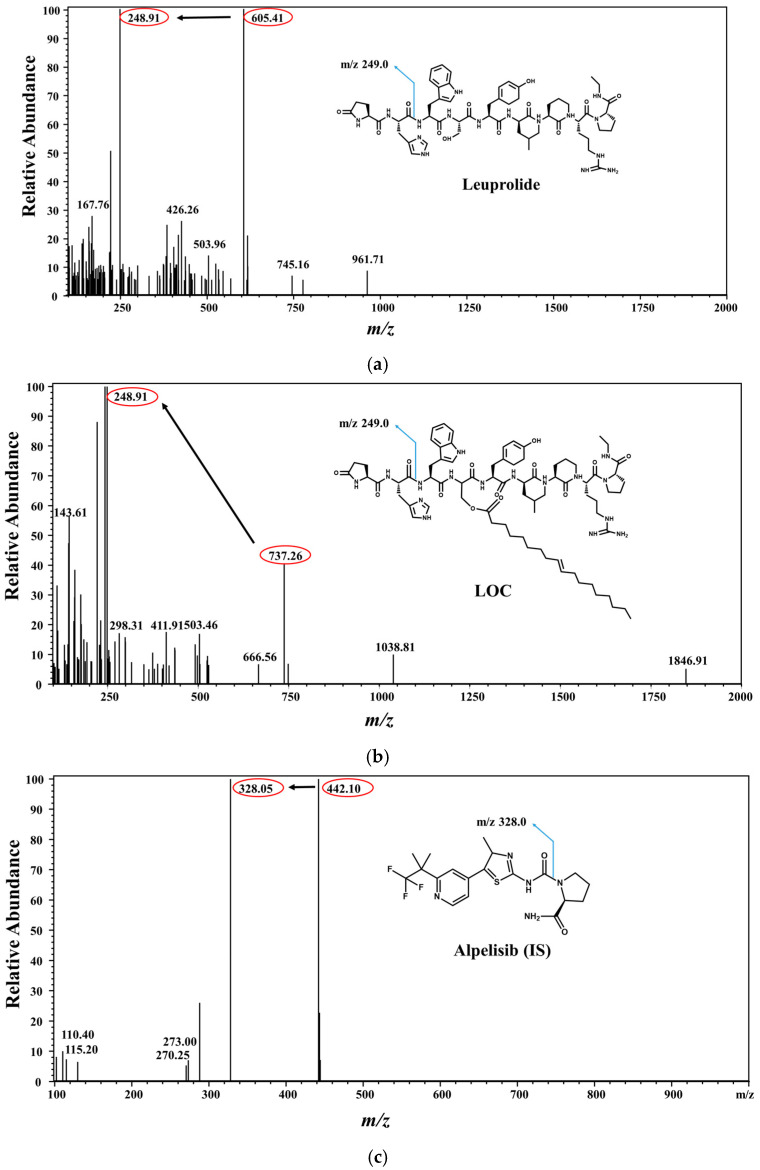
Chemical structures of leuprolide, leuprolide-oleic acid conjugate (LOC), and alpelisib (IS). Mass-product ion-scan spectra and chromatograms of (**a**) leuprolide, (**b**) LOC, and (**c**) IS.

To optimize the electrospray ionization (ESI) conditions for leuprolide, LOCs, and alpelisib (IS), quadrupole full scans were carried out in both positive and negative ion-detection modes, and a good response was achieved in the positive ion mode. In the Q1 full-scan mode, the protonated precursors [M + H]^2+^ of leuprolide and the LOC were *m*/*z* 605.41 and 737.26, respectively. The protonated precursor [M + H]^+^ of IS was *m*/*z* 442.10 (Figure 1). In the Q3 scan mode, the ions at *m*/*z* 248.91, 248.91, and 328.05 were selected as product ions of leuprolide, the LOC, and IS, respectively. Therefore, the ion transitions monitored for quantification were *m*/*z* 605.41→248.91 for leuprolide, 737.26→248.91 for the LOC, and 442.10→328.05 for IS. The collision energies were −29 eV for leuprolide, −37 eV for the LOC, and −35 eV for IS. Formic, acetic, trifluoroacetic, and ammonium acids were tested as mobile phases to optimize separation conditions. A mobile phase made of 0.1% formic acid:ACN facilitated good leuprolide and LOC retention and sensitivity. Several analytical columns, including Poroshell 120 EC-C18 column (100 mm × 2.1 mm, 2.7 μm; Agilent, Santa Clara, CA, USA), Kinetex C18 column (100 mm × 2.1 mm, 2.6 μm, 100 Å; Phenomenex, Torrance, CA, USA), and Ascentis^®^ Express C18, 2.7 μm high-performance liquid chromatography (HPLC) column (50 mm × 2.1 mm, 2.7 μm; Supelco, Bellefonte, PA, USA), were evaluated using the formic-acid mobile-phase system. The Kinetex C18 column showed potential for an improved separation of leuprolide and the LOC from endogenous interferences than the other columns. The LC conditions were further optimized by evaluating the effect of can content. A satisfactory separation of leuprolide and the LOC from endogenous plasma interferences with an acceptable peak resolution was obtained in gradient mode.

### 2.2. Method Validation

#### 2.2.1. Selectivity and Linearity

**Figure 2:**
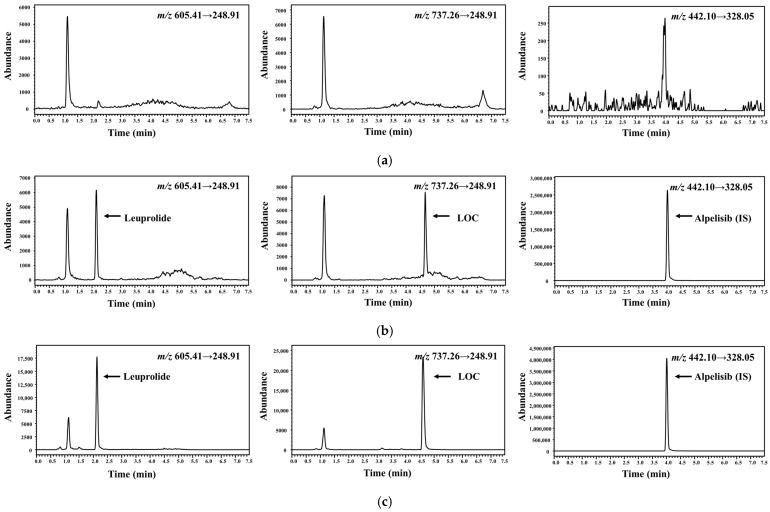
Representative chromatograms of leuprolide, LOC, and IS in rat plasma samples. (**a**) Blank rat plasma. (**b**) Blank rat plasma spiked with analytes [1 ng/mL, lower limit of quantification (LLOQ)]. (**c**) Plasma sample collected at 60 min after subcutaneous administration at a dose of 0.122 mg/kg of LOC (0.1 mg/kg in terms of leuprolide) in rats, wherein the calculated concentrations of leuprolide and LOC were 1.86 and 11.7 ng/mL, respectively.

Representative multiple-reaction monitoring (MRM) chromatograms of the plasma samples are shown in Figure 2; the optimized LC conditions resulted in a retention time of 2.17 min for leuprolide, 4.69 min for the LOC, and 4.02 min for IS. MRM chromatograms clearly indicated that no significant interference from the blank plasma was found at the corresponding retention time of leuprolide, the LOC, and IS. Several acids, such as hydrochloric, acetic, and formic acid, were further tested to improve the efficiency of the sample-preparation procedure. The best recovery and selectivity for leuprolide and the LOC were achieved by adding 300 μL of 5% formic acid to the plasma sample. The linearity of the calibration curve was evident over the concentration range of 1–1000 ng/mL with r^2^ ˃ 0.999 in all validation runs. Representative calibration curves were *y* = 0.0012 × *x* + 0.0002 for leuprolide and *y* = 0.0014 × *x* + 0.0003 for the LOC, where *x* and *y* represent the nominal concentrations of leuprolide and the LOC spiked into blank rat plasma and the peak area ratio of the analyte/IS, respectively. Serum leuprolide concentrations > 1 ng/mL were observed for up to 3 weeks in subjects who subcutaneously received 45 mg of leuprolide [18].

#### 2.2.2. Precision and Accuracy

Intra- and inter-day precision and accuracy of leuprolide and the LOC are shown in Table 1. Precision was ≤12.4% for leuprolide and ≤10.1% for the LOC. Accuracy ranged between 93.0–109% for leuprolide and 94.7–110% for LOC. These results indicate that this analytical method is precise, reliable, and reproducible for the simultaneous quantification of leuprolide and the LOC in rat plasma samples.

#### 2.2.3. Extraction Recovery and Matrix Effect

Recovery and matrix effect data for each analyte are presented in Table 1. The mean extraction recoveries from the stabilized rat plasma for all analytes were within the range of 95.8–105% at the three quality control (QC) levels {low QC [LQC], middle QC [MQC], and high QC [HQC]}. The matrix effects were within the range of 90.6–108% for all analytes in stabilized rat plasma. Our results show that the sample-preparation procedures used herein offer an acceptable matrix effect with good extraction recovery for this bioanalytical method.

#### 2.2.4. Stability

The stability of all analytes was tested using two concentration levels (3 and 800 ng/mL) after exposure to different stability conditions. Recovery values of 90.8–103% for leuprolide and 91.2–107% for the LOC were obtained, with deviations ranging between 2.41–6.45, and 1.24–9.02, respectively (Table 2). These results indicate the stability of the studied drug under given conditions; all error and deviation values were <15%. Moreover, solutions of the three analytes were found to be stable when stored in a refrigerator (−20 °C) for 1 month.

### 2.3. Pharmacokinetic Application

**Figure 3:**
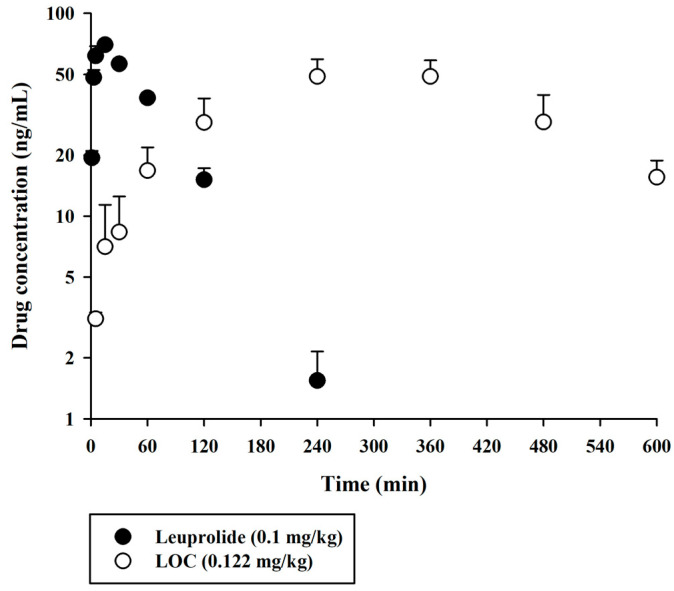
Arterial plasma concentration versus time profiles of leuprolide and the LOC following the subcutaneous administration of leuprolide and the LOC in rats. The closed and open circles and their error bars represent the mean and standard deviation values, respectively (*n* = 4).

**Figure 4:**
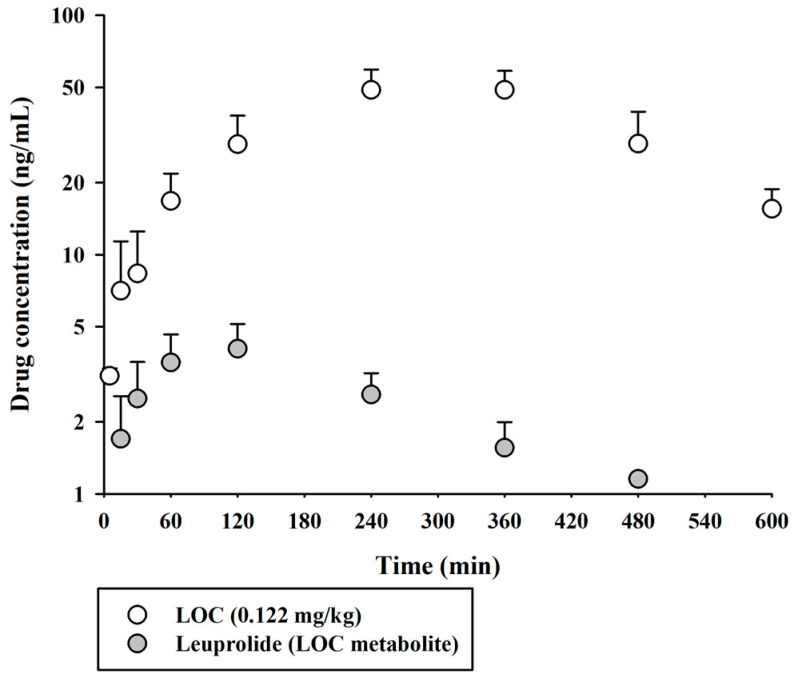
Arterial plasma concentration versus time profiles of the LOC and leuprolide (as a metabolite of the LOC) following the subcutaneous administration of the LOC in rats. The open and closed circles and their error bars represent the mean and standard deviation values, respectively (*n* = 4).

The validated UPLC-MS/MS method was successfully applied to compare the pharmacokinetic behaviors of unmodified leuprolide and the leuprolide-fatty acid conjugate in rat plasma. A single dose of leuprolide and LOC was administered subcutaneously to male SD rats. The mean plasma concentration-time curves of leuprolide and the LOC in a single-dose study of leuprolide and LOC are shown in Figure 3. Figure 4 shows the mean plasma concentration-time curves of the LOC and its metabolite (leuprolide) following the subcutaneous administration of the LOC in rats. The corresponding pharmacokinetic parameters are presented in Table 3. The peak plasma concentration (C_max_) was 70.0 ± 3.7 and 52.9 ± 7.1 ng/mL, the time to reach C_max_ (T_max_) was 15 and 300 min, the area under the plasma concentration–time curve from time zero to the last sampling time (AUC_last_) was 5396 ± 322 and 19,545 ± 2483 ng·min/mL, and the half-life of drug elimination—at the terminal phase (t_1/2_)—was 38.2 ± 4.3 and 172 ± 66 min for leuprolide and the LOC, respectively, after subcutaneous administration. The LOC was slowly absorbed after subcutaneous administration and slowly eliminated from the circulation compared to leuprolide. These results supported the notion that, like other peptides, leuprolide is also susceptible to proteolysis and can be filtered through the glomeruli of the kidneys; therefore, it can be rapidly cleared from the systemic circulation following parenteral administration [14,19]. However, after the subcutaneous administration of the LOC, the t_1/2_ increased by 4.33 times and the mean residence time (MRT_inf_) by increased by 4.36 times compared to that in the unmodified leuprolide group. According to previously reported data for leuprolide, the MRT_inf_ in the intravenous group was calculated as 28.1 min [16]. The mean absorption time (MAT) in the leuprolide and LOC groups was estimated to be 36.8 and 255 min, respectively. When leuprolide was conjugated with oleic acid, the MAT increased by approximately 6.93 times compared to that of unmodified leuprolide. Conjugation with a fatty acid chain increased the molecular weight, hydrophobicity, and hydrodynamic volume of the peptide drug, which could change the absorption and renal clearance of the drug. The attached fatty acid chain may protect the peptides from proteolytic degradation. Overall, the circulation time of the peptide drug was prolonged.

## 3. Materials and Methods

### 3.1. Materials and Animals

Leuprolide acetate (used as the drug) was purchased from Anygen (Gwangju, Republic of Korea). Lorelin injection^®^ was obtained from Dongkook Pharmaceutical (Seoul, Republic of Korea). Oleic acid (used as a fatty acid) was purchased from Sigma-Aldrich (St. Louis, MO, USA). Alpelisib (purity > 99%) was purchased from MedKoo Bioscience Inc. (Morrisville, NC, USA). HPLC-grade acetonitrile and methanol were purchased from Honeywell Inc. (Muskegon, MA, USA). Heparin injectable solution was purchased from Huons Co. (Jecheon, Republic of Korea). Eight-week-old male SD rats (body weight, 240–250 g) were purchased from Koatech Co. (Incheon, Republic of Korea). The protocol in this study was performed according to the guidelines of the Institutional Animal Care and Use Committee of Pusan National University and was reviewed and approved on 15 April 2021 (Busan, Republic of Korea; approval number: PNU-2022–3190).

### 3.2. Synthesis of LOC

The LOC was synthesized by conjugating oleic acid with the hydroxyl group of leuprolide acetate using benzoyl chloride and 4-dimethylaminopyridine via the Yamaguchi esterification method [20]. The experimental setups were described in detail in our previous paper [17].

### 3.3. LC-MS/MS Conditions

Chromatographic analysis was performed on a Shimadzu Nexera LC-30AD UPLC system, and mass spectrometric detection was performed on an LCMS-8050 triple quadrupole mass spectrometer (Shimadzu Co., Kyoto, Japan) in the positive ESI and MRM mode. The liquid chromatographic separation of leuprolide, the LOC, and IS (alpelisib) was conducted at 30 °C using the Kinetex XB-C18 column (100 mm × 2.1 mm, 2.6 μm; Phenomenex, Torrance, CA, USA), protected by a C18 guard column (SecurityGuard ULTRA; Phenomenex, Torrance, CA, USA). The gradient elution of the mobile phase consisting of 0.1% formic acid in water (solvent A) and 0.1% formic acid in acetonitrile (solvent B) was performed at a flow rate of 0.3 mL/min as follows (solvent A:solvent B, *v*/*v*): maintained at 19.3 for 0.01 min; ramped from 19.3 to 39.6 for 2.49 min; maintained at 39.6 for 0.01 min; ramped from 39.6 to 90 for 2.99 min; reverted to 19.3 for 0.01 min; and maintained for 1.99 min (total run time: 7.5 min). The ion source parameters were set as follows: nebulizing gas flow, 3 L/min; drying gas flow, 10 L/min; heating gas flow, 10 L/min; interface temperature, 300 °C; desolvation temperature, 250 °C; and heating block temperature, 400 °C.

### 3.4. Calibration Standard and QC Samples

Stock solutions of leuprolide, the LOC, and IS were prepared at a concentration of 1000 μg/mL in methanol. These solutions were serially diluted using methanol to prepare working standard solutions of 0.1–100 µg/mL. The working IS solution was prepared at a concentration of 2 μg/mL in acetonitrile. Blank rat plasma was spiked with each working standard solution to obtain final plasma concentrations of 1, 2, 5, 10, 20, 50, 100, 200, 500, and 1000 ng/mL as calibration standard samples. QC samples were prepared using the same process as that used for the calibration standards. QC levels of leuprolide and the LOC were set at 1 ng/mL for LLOQ, 3 ng/mL for LQC, 75 ng/mL for MQC, and 800 ng/mL for HQC.

### 3.5. Sample Preparation

Plasma samples (100 µL) were mixed with 300 µL of 5% formic acid (in water) and vortexed for 5 min. Then, 500 µL acetonitrile of containing 20 ng/mL IS was added to the resultant mixture and vortexed for 5 min. After centrifugation at 14,500× *g* for 10 min at 4 °C, 800 µL of the supernatant was collected, dried under vacuum using a SpeedVac (Eyela, Tokyo, Japan), reconstituted with 50 μL of methanol, and injected into the LC-MS/MS system.

### 3.6. Method Validation

Using the calibration standard and QC samples, the present LC-MS/MS method was validated in terms of its selectivity, LLOQ, linearity, accuracy, precision, recovery, and stability according to the United States Food and Drug Administration guidelines for bioanalytical method validation [20]. Selectivity was evaluated by comparing the chromatograms of blank rat plasma samples, spiked blank rat plasma samples, and rat plasma obtained from rat pharmacokinetic studies, followed by checking for the presence of potential interferences at the leuprolide-, LOC-, and IS-acquisition windows. Linearity was determined by 10 standards over a concentration range of 1–1000 ng/mL. Linearity for leuprolide and the LOC was plotted using the peak area ratio (drug/IS) versus concentration. For LLOQ, the signal-to-noise (S/N) ratio was required to be greater than or equal to 10. Precision and accuracy were assessed in replicates of five at four QC levels on five successive days, each with an independently prepared calibration curve. Precision was expressed as relative standard deviation, which should not exceed 15%, while accuracy was expressed as a relative error, which had to be within ±15%. The extraction recovery of leuprolide and LOCs from rat plasma was determined by comparing the peak area of the analyte in the extracted QC samples (LQC, MQC, and HQC) with the peak area of the analyte reconstituted in the blank rat plasma extract at the same concentration. The matrix effect was determined by comparing the peak area of the analyte reconstituted in the blank rat plasma extract with that of standard solution at the corresponding concentration. The value of the matrix effect should be in the range of 85–115%. If one depicts the peak areas obtained in neat solution standards as *A*, the corresponding peak area for standards spiked after extraction into plasma extract as *B*, and peak areas for standards spiked before extraction as *C*, the recovery and matrix effect value can be calculated as follows:

$$\mathrm{Matrix}\mathrm{effect}\left(\%\right)=\frac{B}{A}\times 100$$

$$\mathrm{Recovery}\left(\%\right)=\frac{C}{B}\times 100$$

The extraction recovery and matrix effect were assessed in five replicates at four QC levels. Stability was determined under various analytical handling and storage conditions, such as bench-top, freeze–thaw, post-preparative, and long-term storage, at two QC levels (LQC and HQC).

### 3.7. In Vivo Pharmacokinetic Study of Rats

Rats underwent surgical implantation of a cannula in the femoral artery under anesthesia, as previously described [21,22,23]. The LOC was dissolved in phosphate-buffered saline (pH 5.7). Different leuprolide formulations (Lorelin injection^®^ and LOC) were subcutaneously injected at doses of 0.1 mg/kg for leuprolide and 0.122 mg/kg (0.1 mg/kg as leuprolide) for the LOC into rats. A blood sample of approximately 0.3 mL was collected via femoral artery cannulation at various time points (0, 1, 3, 5, 10, 20, 30, 60, 90, 120, 180, 240, 360, 480, and 600 min), then collected in heparin pre-treated microcentrifuge tubes. These blood samples were centrifuged at 4000× *g* for 10 min to obtain plasma and were frozen at −20 °C until analysis.

### 3.8. Pharmacokinetic and Statistical Analyses

Non-compartmental analysis was performed using Phoenix WinNonlin ver. 3.1 (Certara USA Inc., Princeton, NJ, USA) to estimate the following pharmacokinetic parameters: AUC_last_; total area under the plasma concentration versus time curve from time zero to time infinity (AUC_inf_); C_max_; T_max_; t_1/2_; and MRT_inf_. Values were obtained directly from the concentration–time plot. All values are reported as the mean ± standard deviation. Statistical significance was defined as a statistical value < 0.05, which was estimated by *t*-test for the comparison of the two unpaired means.

## 4. Conclusions

In conclusion, a simple UPLC-MS/MS method was successfully developed and validated for the simultaneous quantitative determination of leuprolide and LOCs in rat plasma. The developed method offers several advantages, such as the ease of sample preparation, good recovery, negligible matrix effect, and a wide assay range that covers serum leuprolide concentrations observed in clinical settings. In vivo rat studies revealed that the pharmacokinetic properties of LOCs, a fatty acid conjugate, were improved compared to those of leuprolide. Fatty acid conjugates displayed slow absorption and a prolonged circulating half-life. To the best of our knowledge, this is the first study to show the effects of fatty acid conjugates on the pharmacokinetics of leuprolide using a newly developed UPLC-MS/MS method for the simultaneous quantification of leuprolide and LOCs.

## Figures and Tables

**Table 1:** Precision, accuracy, recovery, and matrix effect of liquid chromatography-electrospray ionization–tandem mass spectrometry (LC-ESI-MS/MS) analysis of leuprolide and leuprolide-oleic acid conjugate (LOC) in rat plasma samples (*n* = 5).

Nominal Concentration (ng/mL)	Precision (%)		Accuracy (%)		Recovery (%)	Matrix Effect (%)
	Intra-Day	Inter-Day	Intra-Day	Inter-Day		
*Leuprolide*						
Lower limit of quantification (LLOQ) (1)	≤12.4	≤6.92	104 ± 13	98.1 ± 6.8		
Low quality control (LQC) (3)	≤4.04	≤8.33	109 ± 4	99.8 ± 8.3	105 ± 2	108 ± 4
Medium quality control (MQC) (75)	≤3.21	≤7.44	107 ± 3	97.0 ± 7.2	105 ± 5	104 ± 7
High quality control (HQC) (800)	≤6.10	≤4.90	99.3 ± 6.0	93.0 ± 4.6	103 ± 4	106 ± 9
*LOC*						
LLOQ (1)	≤10.1	≤4.74	110 ± 11	103 ± 4.9		
LQC (3)	≤1.36	≤4.35	100 ± 1	102 ± 4.4	99.2 ± 3.9	96.7 ± 3.4
MQC (75)	≤5.15	≤5.78	97.4 ± 5.0	97.5 ± 5.6	95.8 ± 6.1	95.0 ± 5.6
HQC (800)	≤5.96	≤7.04	94.7 ± 5.6	97.7 ± 6.9	98.8 ± 3.8	90.6 ± 4.1

**Table 2:** Stability (as the percentage of drug remaining) of leuprolide and leuprolide-oleic acid conjugate (LOC) in rat plasma samples (*n* = 5).

Nominal Concentration (ng/mL)	Bench-Top ^1^	Autosampler ^2^	Freeze–Thaw ^3^	Long-Term ^4^
*Leuprolide*				
LQC (3)	96.5 ± 2.4	103 ± 6	92.6 ± 4.5	91.1 ± 4.4
HQC (800)	97.4 ± 3.7	101 ± 4	90.8 ±2.5	96.3 ± 2.5
*LOC*				
LQC (3)	105 ± 7	102 ± 9	107 ± 4	91.2 ± 4.0
HQC (800)	97.7± 2.0	92.7 ±1.2	107 ± 4	93.4 ± 2.6

^1^ Room temperature for 3 h. ^2^ 10 °C for 24 h in the autosampler. ^3^ Three freezing and thawing cycles every 12 h at −20 °C. ^4^ −20 °C for 30 d.

**Table 3:** Pharmacokinetic parameters of leuprolide and the LOC following their subcutaneous injection in rats (*n* = 4).

Parameter	Leuprolide Group	LOC Group
	*Leuprolide*	*LOC*
Total area under the plasma concentration versus time curve from time zero to time infinity (AUC_inf_) (ng·min/mL)	5484 ± 364	23,581 ± 4068
The area under the plasma concentration–time curve from time zero to the last sampling time (AUC_last_) (ng·min/mL)	5396 ± 322	19,545 ± 2483
Half-life of drug elimination at the terminal phase (t_1/2_) (min)	38.2 ± 4.3	172 ± 66
Peak plasma concentration (C_max_) (ng/mL)	70.0 ± 3.7	52.9 ± 7.1
Time to reach C_max_ (T_max_) (min)	15	300 (240–360)
Mean residence time (MRT_inf_) (min)	64.9 ± 4.6	405 ± 64
		*Leuprolide* *after administration of LOC*
AUC_inf_ (ng·min/mL)		1634 ± 328
AUC_last_ (ng·min/mL)		1265 ± 317
t_1/2_ (min)		166 ± 38
C_max_ (ng/mL)		4.9 ± 1.3
T_max_ (min)		120
MRT_inf_ (min)		283 ± 53

## Data Availability

Not applicable.
